# A Material Combination Concept to Realize 4D Printed Products with Newly Emerging Property/Functionality

**DOI:** 10.1002/advs.201903208

**Published:** 2020-03-20

**Authors:** Hongzhi Wu, Xuan Zhang, Zheng Ma, Ce Zhang, Jingwei Ai, Peng Chen, Chunze Yan, Bin Su, Yusheng Shi

**Affiliations:** ^1^ State Key Laboratory of Materials Processing and Die and Mould Technology School of Materials Science and Engineering Huazhong University of Science and Technology Wuhan 430074 P. R. China; ^2^ State Key Laboratory of Advanced Electromagnetic Engineering and Technology Huazhong University of Science and Technology Wuhan 430074 P. R. China

**Keywords:** 4D printing, additive manufacturing, flexible magnetoelectronics, material combination, piezoelectronics

## Abstract

4D printing is a newly emerging technique that shows the capability of additively manufacturing structures whose shape, property, or functionality can controllably vary with time under external stimuli. However, most of the existing 4D printed products only focus on the variation of physical geometries, regardless of controllable changes of their properties, as well as practical functionality. Here, a material combination concept is proposed to construct 4D printed devices whose property and functionality can controllably vary. The 4D printed devices consist of conductive and magnetic parts, enabling the integrated devices to show a piezoelectric property even neither part is piezoelectric individually. Consequently, the functionality of the devices is endowed to transfer mechanical to electrical energy based on the electromagnetic introduction principle. The working mechanism of 4D printed devices is explained by a numerical simulation method using Comsol software, facilitating further optimization of their properties by regulating diverse parameters. Due to the self‐powered, quick‐responding, and sensitive properties, the 4D printed magnetoelectric device could work as pressure sensors to warn illegal invasion. This work opens a new manufacturing method of flexible magnetoelectric devices and provides a new material combination concept for the property‐changed and functionality‐changed 4D printing.

## Graphical abstract

This work proposes a material combination concept, by combining additively manufactured magnetic and electrical building blocks, to construct new 4D printed products. The integrated 4D printed devices show a controllably changed piezoelectric property under the exerted external pressure, as well as new functionality of working as self‐powered pressure‐sensitive monitors.

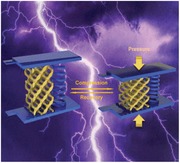

## 1 Introduction

Additive manufacturing (AM) including 3D printing and 4D printing technologies creates a physical object by depositing materials, layer upon layer, based on a digital model.^[^ ^1^, ^2^ ^]^ Traditional 3D printing can generate complex‐structural products in one step, and however fails to endow the products with a “smart” feature. Therefore, 4D printing emerged in 2013, which was initially defined as “3D printing + time,” and only the shape of printed products can change over time. Soon after that, the concept of 4D printing has evolved into a reliable definition: 4D printing is a technique that shows the capability of additively manufacturing structures whose shape, property, or functionality can controllably vary with time under external stimuli.^[^ ^3^ ^]^ Consequently, 4D printed products can make functional adjustments according to the changes in the external environment, which will lead to a wide variety of applications in energy, military, aerospace, biomedicine, soft robotics, and electronic devices.^[^ ^4^ ^−^ ^8^ ^]^

Stimulated by the promising prospect, strenuous efforts have been made to explore various strategies for 4D printing by demonstrating shape memory effect (SME) of 3D printed structure. Qi et al. pioneered the printed active composite materials^[^ ^9^, ^10^ ^]^ shifting into an intricate configuration directly by heating. Another important strategy is to use shape memory materials (SMM),^[^ ^11^ ^−^ ^14^ ^]^ mainly including shape memory polymers (SMPs)^[^ ^15^ ^−^ ^19^ ^]^ and shape memory alloys (SMAs)^[^ ^20^, ^21^ ^]^ in AM. As‐prepared products can change from their temporary structures to the initial configurations when exposed to a high temperature environment. Besides motivated by heat, in recent years, 4D printed structures driven by electricity,^[^ ^22^, ^23^ ^]^ magnetism,^[^ ^24^ ^−^ ^26^ ^]^ pH,^[^ ^27^, ^28^ ^]^ solvent,^[^ ^29^ ^−^ ^31^ ^]^ light,^[^ ^32^, ^33^ ^]^ and biological signals^[^ ^34^ ^]^ have also appeared. Although tremendous efforts have been paid in this field, all above‐mentioned 4D printed matters can only show shape‐changing demonstrations, regardless of their changes in properties and the functionality. It is worth noting that the changes in property and functionality are more important in 4D printing since they will greatly promote its practical applications in future. However, it is difficult to achieve controllable changes in the property/functionality of 4D printed products by merely using shape‐shift smart materials in the AM process. Thus, it is highly challengeable and urgent to seek an effective way to implement property‐changed and functionality‐changed 4D printing.

**Figure 1:**
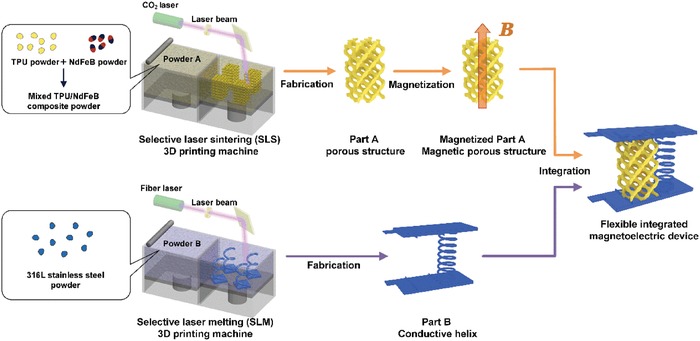
Schematic illustration of the fabrication process of the flexible integrated magnetoelectric device assembled by parts A and B. The part A, a porous structure, was fabricated by selective laser sintering (SLS) additive manufacturing (AM) process using TPU/NdFeB (where, TPU, thermoplastic polyurethane) composite powders and then was magnetized to acquire permanent magnetism. The part B, a helix structure with two flat plates, was fabricated by selective laser melting (SLM) AM process using 316L stainless steel powders.

Different from existing methods by using shape‐shift smart materials to realize 4D printing, we propose a material combination concept, by integrating additive manufactured magnetic and conductive building blocks (**Figure** **1**), to construct 4D printed devices based on our previous studies.^[^ ^35^ ^]^ Owing to the electromagnetic introduction principle between magnetic and conductive parts, the integrated devices would show a piezoelectric property even neither part is piezoelectric individually, indicating the change of their property. Accordingly, the functionality of the integrated 4D printed devices can be improved to transfer mechanical to electrical energy. Consequently, the material combination concept will lead to the property and functionality change of as‐prepared 4D printed devices.

To fulfill this concept regarding 4D printing, herein we reported flexible integrated magnetoelectric devices enabling the mechanical pressure to be converted to electrical signals. The newly emerging self‐powered piezoelectric property exhibits a controllable variation under the applied pressure. In addition, the as‐prepared devices show a new functionality of working as pressure‐sensitive monitors. The working mechanism can be theoretically explained by numerical simulation, facilitating further optimization of their properties by tuning magnetic powder content, the direction of the magnetic field, compression rate, and other factors. Due to their self‐powered piezoelectrical properties, these flexible magnetoelectric devices can serve as intelligent pressure sensors to warn illegal invasion. This work lays the foundation for material and structure design of 4D printed devices that exhibit new property and functionality besides the shape changes. It will expand myriads of functional applications such as flexible sensors in the field of 4D printing.

## 2 Results and Discussion

The AM processes of selective laser sintering (SLS) and selective laser melting (SLM) were employed to fabricate integrated 4D printed devices consisting of a magnetic porous structure (part A) and a conductive helix structure connected with two flat plates (part B), as shown in Figure 1. The part A was built by SLS using the composite powders of thermoplastic polyurethane (TPU, Figure S1d, Supporting Information) and NdFeB (Figure S1c, Supporting Information). The NdFeB powders have permanent magnetism with the residual flux density (*B*_r_) and maximum magnetic energy product (*BH*_max_) being 787.50 mT and 97.024 kJ m^−3^. The NdFeB particles were mixed with the TPU powders to obtain average particle sizes around 50 µm (Figure S1e, Supporting Information), which were suitable for the SLS process. The TPU is a flexible material with a low compression modulus, allowing a well compressed capacity for the 4D printed devices.

**Figure 2:**
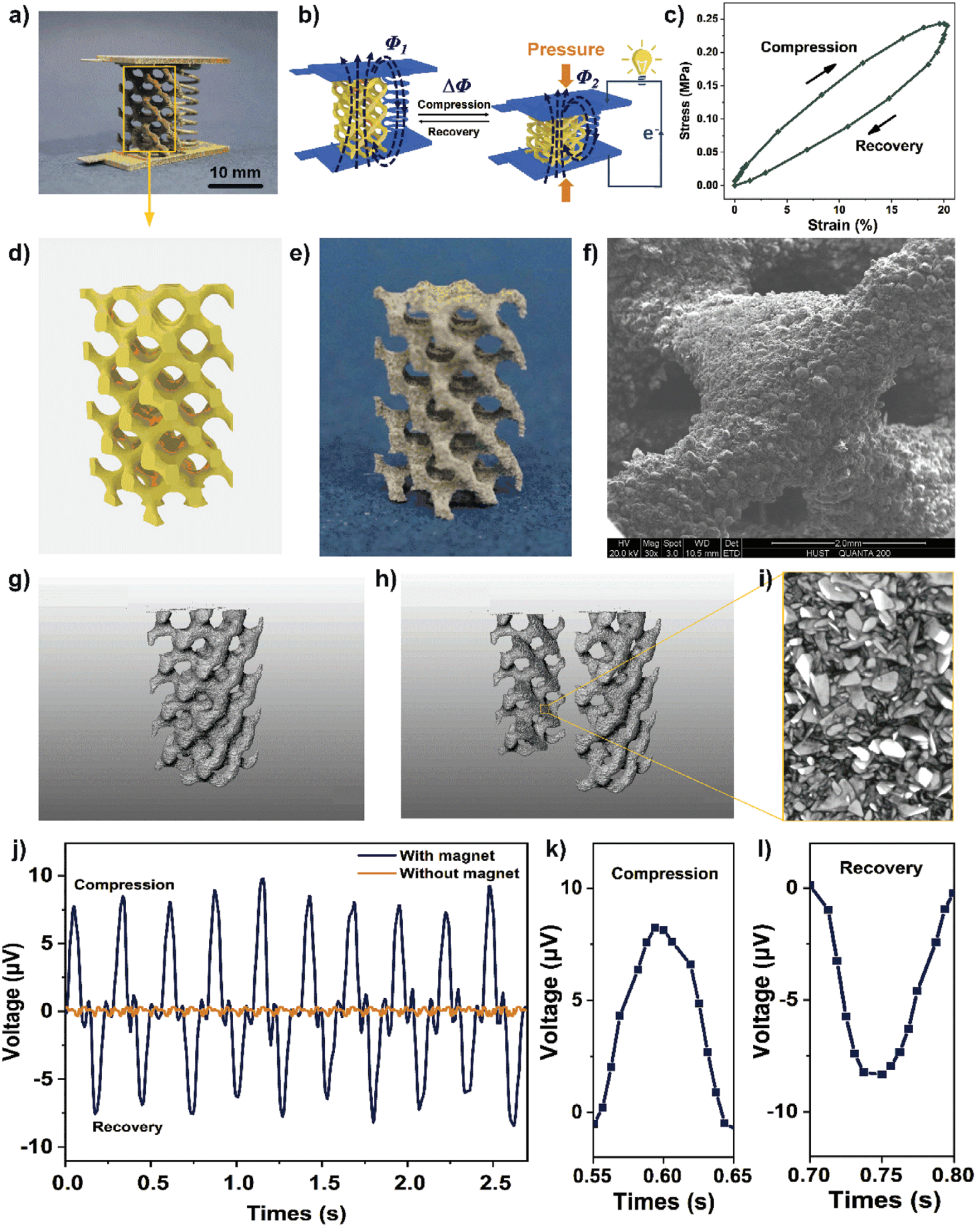
As‐prepared flexible magnetoelectric devices exhibit a piezoelectric ability that can convert external pressure to electrical outputs. a) Optical photograph of a flexible integrated magnetoelectric device with a height of 25 mm. b) Schematic diagram showing integrated 4D printed devices that can generate electrical outputs. This is because the magnetic flux in the helix structure changes during the compression/recovery process. c) The correlation of stress and strain of a magnetoelectric device in one cycle at a compression rate of 50 mm s^−1^. d) The computer‐aided design (CAD) model of the magnetic porous structure with the outer cube contour size 25 × 12.5 × 12.5 mm^3^. e) Optical photograph of the porous structure fabricated by selective laser sintering (SLS) process. f) The scanning electron microscope (SEM) images of the surface of the porous structure. g) The 3D micro‐CT image of the magnetic porous, h) its vertical section, and i) the magnetic NdFeB powder in the vertical section. j) The open‐circuit output voltage of a flexible magnetoelectric device by applying a compression strain of 20% and a speed of 50 mm s^−1^. The blue and orange plots denote the output voltage with and without magnetic NdFeB powder in the porous structure. k,l) The output voltage during a single compression cycle.

**Figure** **2**a,e shows the photographs of magnetic porous structure (part A) with a 40 wt% NdFeB content and a size of 25 × 12.5 × 12.5 mm^3^. Figure 2d shows its 3D computer‐aided design (CAD) model. For detailed observation, an SEM image of the porous structure can be found in Figure 2f, showing the joint part of the printed TPU/NdFeB networks. It is easy to observe that NdFeB powders were uniformly dispersed over the TPU‐matrix scaffold. Besides SEM investigation, we also employed the in‐site micro‐CT technique to investigate the inside distribution of NdFeB powders in the TPU scaffolds. Figure 2g,h and Figure S2, Supporting Information, are the porous TPU/NdFeB structures before and after compression, respectively. The white points with brighter color represent the NdFeB powders, indicating that the printed TPU structures were uniformly filled with NdFeB powders.

After magnetization, the SLS‐printed magnetic porous structures with various NdFeB contents (20, 30, and 40 wt%), magnetic field directions (vertical and horizontal), and heights (25, 20, and 15 mm) showed different magnetic induction intensity (Figure S3, Supporting Information). The magnetic induction intensity mainly depends on the NdFeB powder content. The higher the content of NdFeB, the stronger is the magnetic field strength. If the magnetic powder content is excessively increased (>40 wt%), the amount of the TPU will be too low to fabricate the porous structure with enough mechanical properties. The experiments confirmed that the porous structure failed to be fabricated when the magnetic powder content rose to 50 wt%. The magnetic field intensity is about 11.1 mT on the north/south pole of a 40 wt% NdFeB magnetic porous structure. The magnetic field strength at the vertical and horizontal edges of porous structure shows significant difference. This is due to the different density of magnetic lines in the two directions after magnetization.

In the meantime, the part B was fabricated by SLM of the 316L stainless powder (Figure S1a,b, Supporting Information). In the CAD model (Figure S4a, Supporting Information) of the part B, we designed a pair of slots connected by a helix structure to position the above‐mentioned porous TPU/NdFeB structures (part A). In addition, two designed contact chips were connected to the edges of the two plates, allowing integrated 4D printed devices to be expediently plugged into a circuit for further electrical testing. The SLM‐printed part B with seven‐layered coils and a height of 25 mm was shown in Figure 2a and Figure S4b, Supporting Information.

Figure 2a shows the optical photograph of integrated 4D printed devices combined by the magnetic porous structure (part A) and the conductive helix structure (part B). Owing to the porous/helix design of printed structures and elasticity of TPU materials, the combined magnetoelectric device can be easily compressed and then quickly recover to the original shape. This can be proved by the relationship curve between stress and strain (Figure 2c), exhibiting a phase lag loop where the compression curve stays above the recovery curve. When the device was applied a strain of 20%, the stress was 0.24 MPa and the elastic modulus was 1.3 MPa, confirming that the 4D printed structures have good flexibility.

Different from early reported 4D printed products that convert thermal to mechanical energy,^[^ ^10^ ^−^ ^21^ ^]^ the as‐prepared 4D printed device in this study displayed a piezoelectric ability to convert external mechanical pressure to electrical outputs though neither of its parts is piezoelectric. Owing to the existence of magnetic TPU/NdFeB structures, the magnetic field lines would pass through the conductive coil next to the magnetic part. During the compression/recovery cycle driven by external forces, the magnetic flux in the coils would change accordingly, leading to the electric generation based on the electromagnetic induction principle (Figure 2b). In this concept, the property, as well as functionality, of printed devices changed under the exerted external pressure, exhibiting a concept advance of 4D printing.

Regular voltage outputs (Figure 2j) could be observed when exerting a periodic compression on integrated 4D printed devices. The average voltage value (blue plots) is around 8.4 µV at a compression speed of 50 mm s^−1^ and a compression ratio of 20%. For comparison, a control group consisting of a porous structure without magnetic powders was also studied. It was found that none of electric response appeared (orange plots) under the same compression conditions, showing that the magnetic material played a key role in the generation of the piezoelectric effect. Figure 2k,i are enlarged pictures of Figure 2j in one compression/recovery cycle, respectively. The electrical signal curve with periodic upward and then downward shapes is clearly found, exhibiting a peak/valley feature in each compression/recovery cycle.

**Figure 3:**
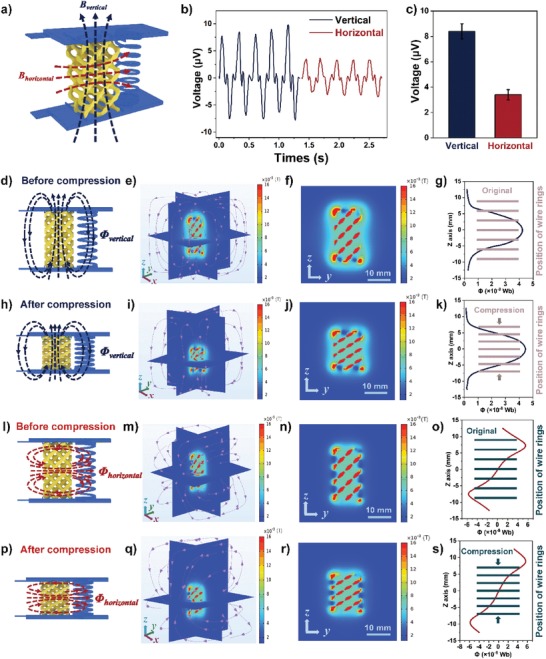
The flexible magnetoelectric device gets different voltage outputs when changing magnetic field direction (take vertical and horizontal as examples) of the porous structure. a) The schematic illustration of two types of magnetic field directions, vertical (blue) and horizontal (red). b) The measured output voltage of a magnetoelectric device under the two magnetic field directions and under same experimental conditions. c) The histogram of output voltage when the magnetic field direction is vertical and horizontal, respectively. d) The schematic illustration of the magnetic field distribution of a magnetoelectric device before pressure is applied when the porous structure is magnetized vertically. The blue dotted lines with arrows indicate the direction of the magnetic field from the S to N pole. e) 3D and f) 2D (*y*−*z* plane, that is, the central plane perpendicular to the *x*‐axis) calculated distribution of the magnetic field intensity of the porous structure. The purple solid lines with arrows in the 3D illustration in (e) represent the magnetic line. g) The dependence of the magnetic flux (Φ) on the *z*‐axis distance. The seven horizontal lines are a simplified representation of the position of the coil (the conductive helix). The pictures (d–g), (h–k), (l–o), and (p–s) can be divided into four similar groups. Different from the aforementioned picture (d–g), the (h–k) represents the device is magnetized vertically after compression, while the (l–o) and (p–s) represent the device is magnetized horizontally before and after compression, respectively.

The direction of the magnetic field can tune the value of magnetic flux through the coil, and thus we further studied the influence of the magnetic field direction (vertical/horizontal) on the output voltage. **Figure** **3**a is a schematic illustration of the porous TPU/NdFeB structure magnetized vertically (blue dotted line) and horizontally (red dotted line). Integrated magnetoelectric devices with diverse magnetic field directions could output different voltages, as shown in Figure 3b. When the porous structure got a horizontal magnetization, the output voltage dropped to only 3.4 µV (Figure 3c), which was reduced by 60% in contrast with that of vertical magnetization.

The emergence of electric generation performance was due to the unique design of materials and structures, and it can be explained by numerical simulation via Comsol software. Figure 3d,l are schematic diagrams of 4D integrated devices consisting of a porous structure with the same NdFeB content (40 wt%) yet different (vertical/horizontal) magnetization directions. Figure 3h,p are their statuses after compression. The 3D simulation models of Figure 3d,h,l,p are shown in Figure 3e,i,m,q, respectively. The simulation models exhibit the 3D distribution of magnetic induction line (purple solid lines with arrows pointing from N pole to S pole) and magnetic field intensity within and around the porous structure. Different colors, from blue to orange in the multicolored strip, signify gradually increasing magnetic intensities.

For easy comparison, 2D illustration (*z*−*y* plane, see Figure 3f,j,n,r) is obtained based on the 3D simulation models. In the outside space of the porous structure, the magnetic intensity shows its strongest value on the top and bottom edges of the porous structure (N pole and S pole). The farther away from the porous structure, the weaker is the magnetic intensity. The calculated magnetic intensities are well coincident with their experimental data, shown in Figure S3, Supporting Information.

To calculate the magnetic flux passing through the conductive coil, the seven‐layered helix structure was simplified equivalently to seven equidistant parallel rings (pink horizontal lines in Figure 3g,k and green horizontal lines in Figure 3o,s). Consequently, the total magnetic flux change of integrated 4D printed devices before and after compression can be calculated applying the following Equation (1)^[^ ^36^ ^]^

(1) $$E(V)=-N•\frac{\Delta \Phi }{\Delta t}=-\sum_{i=1}^{7}\frac{\Delta \Phi i}{\Delta t}=-\sum_{i=1}^{7}\frac{\Phi i(after)-\Phi i(before)}{\Delta t}$$

where *E*(V) is the generated voltage, *N* is the number of rings, ΔΦ is the change in total magnetic flux, ΔΦ*_i_* is the magnetic flux change in each ring, and Δ*t* is the compression time of integrated 4D printed devices. The minus sign denotes the direction of the induced electromotive force according to Lenz's law: the direction of magnetic flux produced by the induced current is always opposed to the change of the initial magnetic flux.

The correlations of *z*‐axis distance and magnetic flux at each continuous height position of the helix structure were calculated. When the porous structure has a vertical magnetic field direction, the calculated blue correlation curves are shown in Figure 3g (before compression) and k (after compression), displaying a shape of normal distribution with the half‐height of the helix structure as the symmetry axis. Thus, we could figure out that the total magnetic flux before and after compression is 14.1 × 10^−8^ Wb (Φ_before_) and 20.4 × 10^−8^ Wb (Φ_after_), respectively. As a result, the total magnetic flux change is 6.3 × 10^−8^ Wb (ΔΦ) (the numerical calculation details can be found in Note S1, Supporting Information). The compression time was about 0.1 s (Figure S7a,b, Supporting Information), and thus the theoretically calculated voltage value could be worked out as 4.4 µV. The difference between the theoretically simulated calculation values and experimental output voltage values mainly attributed to their different essential meanings. The experimental result of 8.4 µV (Figure 2j) is a maximum peak value during the process where integrated 4D printed devices were compressed, and is an instantaneous value in a particular position. In contrast, the theoretical result of 4.4 µV is an average value in the same compression process. Therefore, we should compare the theoretical calculation value of 4.4 µV with the average values in the experiments. Assuming a sinusoidal relationship between voltage and time (the calculation details can be found in Note S2, Supporting Information), as a fitting of the voltage curve (Figure 2j), then the average value was calculated to be 5.3 µV, which is slightly more than the theoretical value of 4.4 µV. This result may attribute to the unavoidable lateral deformation of the helix structure during the compression. Consequently, there is a gap of around 17% between theoretical and experimental results.

In a similar way, if the porous structure was magnetized horizontally, the dependence magnetic flux on the *z*‐axis distances are illustrated as red lines in Figure 3o (before compression) and s (after compression), showing a centrally symmetrical shape centered by the helix structure. The calculated total magnetic flux before and after compression are −1.0 × 1 0^−8^ and 2.0 × 10^−8^ Wb, respectively. As a result, the changed magnetic flux is 3.0 × 10^−8^ Wb (the numerical calculation details can be found in Note S1, Supporting Information) during the 0.1 s compression, yielding a theoretical voltage value of 2.1 µV, which is in good agreement with the average value 2.8 µV (the instantaneous peak value is 4.4 µV) of the experimental results (refer to Note S2, Supporting Information, for details of the calculation).

**Figure 4:**
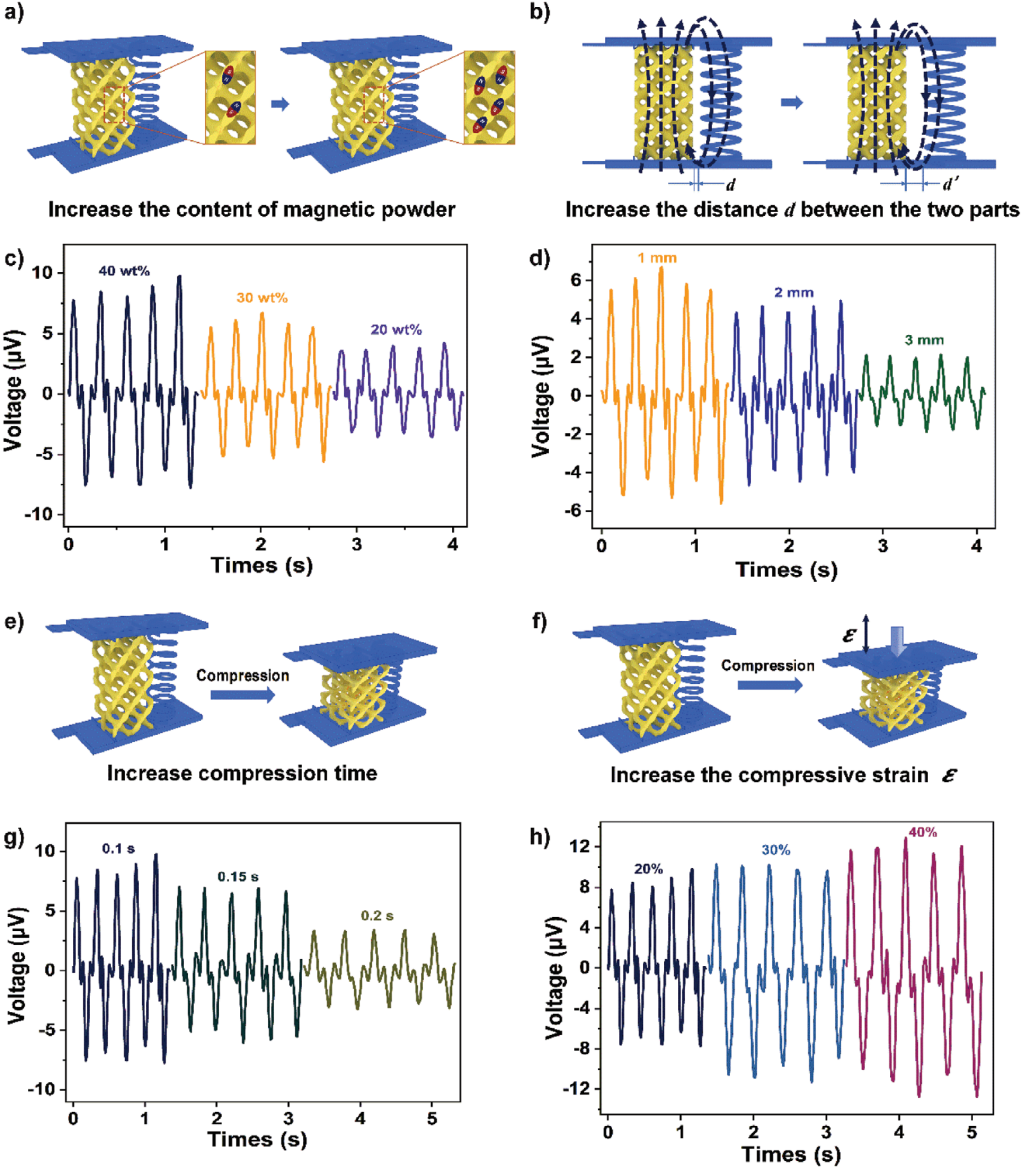
Different fabrication and testing parameters to affect the electrical output of integrated 4D printed devices. The open‐circuit output voltage of integrated 4D‐printed devices manufactured with c) different magnetic powder contents, d) distance (*d*) between the porous structure and the helix structure, g) compression time, and h) compression strains (ε). a,b,e,f) Schematic illustrations of (c,d,g,h), respectively. The other parameters remained the same and only one parameter was tuned to investigate its effect on the electrical properties of integrated 4D‐printed devices.

The influence of material and structure design on piezoelectric properties has been investigated. We increased the weight content of NdFeB powder (from 20 to 40 wt%, see **Figure** **4**a). Following the adding of NdFeB quantity, the magnetic intensity of TPU/NdFeB porous structures increased, resulting in the output voltage enhancement from 3.2 to 8.4 µV (Figure 4c). This can be explained by Faraday's law of electromagnetic induction in terms of Equation (2) as follows

(2) $$E(V)=-\frac{d\Phi }{dt}=-\frac{d}{dt}\int \text{​}\text{​}\text{​}\int B.ds=-\int \text{​}\text{​}\text{​}\int \frac{\partial B}{\partial t}\cdot ds$$

where *B* is the magnetic intensity and *S* is the horizontal area of each ring.

It is obvious that the increase of *B* definitely leads to the rise of the output voltage. Then, taking the integrated devices containing a NdFeB content of 30 wt% as an example, the distance *d* between the porous structure and the helix structure was tailored from 1 to 3 mm, leading to a weaker magnetic flux into the coils (Figure 4b). As a result, the output voltage decreased from 5.7 to 2.0 µV (Figure 4d). Furthermore, the effect of device height has also been studied (Figure S4c,S8, Supporting Information) by tuning the device heights from 25 to 15 mm. The reduction in height leads to the decrease of the magnetic powder content and thus weakens the magnetic induction intensity. Therefore, the output voltage value decreases from 5.7 to 3.1 µV, as shown in Figure S8c, Supporting Information. The number of circles of the helix structure is also reduced when the height decreases. The Figure S8d, Supporting Information, shows the output voltage generated per coil is almost the same 0.8 V even if the height changes.

Diverse measuring parameters, such as the compression rate and strain, have also been studied in detail. We changed the compression rate by adjusting the compression time (Figure 4e). The faster compression rate is equal to the shorter compression time, contributing to the reduction of ∂*t* in Equation (2) Therefore, the output voltage increased, which is consistent with the experimental results (Figure 4g). The larger compression ratio resulted in a higher value of the output voltage, as shown in Figure 4f,h. The output voltage attained 11.8 µV when integrated 4D printed devices were compressed by 40%, since the increased compression strain will enlarge the concentration of NdFeB magnetic powders, thus producing a stronger magnetic flux variation, as well as higher output voltage. The output voltage was enhanced by almost 40% when the compression strain increased from 20% to 40%.

The 4D printed magnetoelectric devices can be used as a self‐powered pressure sensor due to its functionality of converting pressure into an electrical signal. To demonstrate its potential application, as‐prepared devices were buried under the warning line of a virtual bank to warn illegal invasion (Figure S9, Supporting Information). Once gangsters attempt to rob and step on the warning line, integrated 4D printed devices will be compressed, generating an alarm electrical signal timely to inform the police. The schematic diagram of the entire process and the working mechanism of the 4D printed pressure sensors are illustrated in Figure S9a,b, Supporting Information. We selected a “gangster” to do a demonstration (Figure S9c, Supporting Information), the processes of “I→II→III” and “III→IV→V” are two cycles of compression and recovery. The specific description is as follows: (I→II) Integrated 4D printed sensors were compressed from the initial state to the lowest state, simultaneously yielding a peak in the curve of the output voltage. (II→III) As the foot lifted up, integrated 4D printed devices returned to its original state, thereby creating a valley in the output voltage curve. The (IV→V) is a repetitive recovery process. It should be noted that the whole sensors are self‐powered, indicating the energy‐save feature of such 4D printed magnetoelectric sensors.

## 3 Conclusion

In this work, a new type of 4D printing has been implemented by assembling the SLS‐printed magnetic porous structure and SLM‐printed helix structure. Different from shape deformation in the previous 4D printed matters, our integrated 4D printed devices showed a controllably changed piezoelectric property under the exerted external pressure, as well as new functionality of working as self‐powered pressure‐sensitive monitors. The assembled magnetoelectric device can generate electric pulse under the external pressure owing to the magnetic flux changing in the helix, making it available for perceiving pressure. On the basis of the novel design of materials and structures, the change in shape produced by external pressure results in the controllable variation of the electrical generation performance and the emergence of new functionality. Consequently, we have achieved 4D printing of three forms simultaneously containing the change in shape, property, and functionality. As for the 4D printed magnetoelectric devices, several parameters, including the content of NdFeB powders, the direction of the magnetic field, the distance between the porous structure and the helix structure, the compression speed, and the compression ratio, have an effect on the output voltage value. Under an optimal condition, an output voltage of 11.8 µV can be generated by the integrated 4D printed device which is suitable for the smart self‐powered pressure sensors. It is believed that this work may lay the foundation for performance‐changed and functionality‐changed 4D printing by putting forward a material combination concept. We look forward to extending the applications of our 4D printed magnetoelectric device.

## 4 Experimental Section

##### Fabrication of Magnetoelectric Devices

The commercial TPU powder in this work was purchased from Silver Corporation, China. The TPU is one kind of mature commercial products and has good formability in the SLS process. According to the specification, the as‐received TPU powder had a bulk density of 0.50 g cm^−3^. The composite powders with three kinds of NdFeB weight ratios of 20, 30, and 40 wt% were obtained by homogeneously mixing the NdFeB powder, TPU powder, and fumed silica. The fumed silica can improve the fluidity of the composite powders and 1.2 g fumed silica was added to per 100 g of the composite TPU/NdFeB powders. The mixture powders were mixed for 2 min with a mixing rate of 500 revolutions per minute (rpm). Then, the porous structure (Figure 2d, part A in Figure 1) had a unit cell size of 5 mm and a volume fraction of 20% and it was fabricated by the SLS processing of the composite powders using an EOS P 396 machine equipped with a 70 W CO_2_ laser. During the SLS process, the NdFeB powders were not magnetic. Thus, few NdFeB particles could adhere to the metallic blade or the chamber of powder bed caused by magnetic attractive force. The optimized processing parameters characterized by forming chamber temperature, laser power, scan velocity, scan spacing, and layer thickness are summarized in **Table** **1**. After the SLS processing, all the SLS fabricated porous structures were magnetized by a magnetizing apparatus (JIUJU Co., Ltd, China) applying a magnetization voltage of 1900 V. Finally, a magnetic porous structure with a height of 25 mm (Figure 2e) was prepared. For the composite powder with 30 wt% NdFeB, the porous structures with different heights of 20 and 15 mm were also fabricated.

**Table 1:** Optimized selective laser sintering (SLS) processing parameters for the composite powders with three kinds of NdFeB weight ratios

NdFeB weight ratio [wt%]	Preheating temperature [°C]	Laser power [W]	Scan velocity [mm s^−1^]	Scan spacing [mm]	Layer thickness [mm]
20	115	40	4000	0.3	0.12
30	118	40	4000	0.3	0.12
40	120	40	4000	0.3	0.12

As for the conductive helix structure connected with two flat plates (Figure S4, Supporting Information, part B in Figure 1) it was fabricated by the SLM process of a 316L stainless steel powder using a FORWEDO LM200 SLM machine with a 500 W fiber laser. The 316L stainless steel powder was purchased from Forwedo Corporation, China. The optimized processing parameters are shown in **Table** **2**. As a result, the heights of 25, 20, and 15 mm of the helix structure were fabricated. Finally, the 25, 20, and 15 mm high magnetoelectric devices were prepared by assembling the SLS‐printed TPU/NdFeB composite porous structures and the SLM‐printed stainless steel helix structures with the same height.

**Table 2:** Optimized selective laser melting (SLM) processing parameters of the 316L stainless steel powder

Material	Laser power [W]	Scan velocity [mm s^−1^]	Scan spacing [mm]	Layer thickness [mm]
316L	150	1200	0.08	0.04

##### Characterization

A commercial camera (α6300, Sony) was utilized to acquire the optically digital images of the porous structures, the helix structures, and the assembled magnetoelectric devices. The morphological characterization of the TPU powder, NdFeB powder, 316L powder, and surface of the fabricated porous structure was conducted by an environmental scanning electron microscope (ESEM, Quanta 200, FEI, Netherland). The particle sizes and their distributions of the TPU powder, NdFeB powder, and composite powders were measured by a laser particle size analyzer (Mastersizer 3000, Malvern Panalytical, Britain). The magnetic strength on the surface of the porous structure was measured using a WT10A Teslameter (WEITE Magnetic Technology Co., Ltd, China). An electronic dynamic static fatigue testing machine (E1000, Instron‐Division of ITW Limited) was employed to achieve a quantitatively controlled continuous compression/recovery process for magnetoelectric devices and to test the relationship between stress and strain in the cyclic process. The output voltage of integrated 4D printed devices during the compression/recovery process was recorded using a Data acquisition and multimeter system (DMM 6500, Tektronix) with an internal resistance of 1MΩ.

##### Numerical Simulation

COMSOL Multiphysics finite element analysis software was utilized to carry out 3D and 2D modelings of the magnetic porous structures and figure out their magnetic field distribution when magnetized vertically/horizontally and before/after compression. In addition, the magnetic flux and its variation in the helix structure were also calculated. In the calculated model, the permanent magnetic NdFeB powder was assumed to be homogeneously filled in the porous structure, and the magnetic coercivity (*H*_cb_) was set to be 440.03 kA m^−1^ and the residual flux density (*B*_r_) was set to be 787.50 mT. The helix structure considered as conducting coils was simplified into equivalent parallel rings with the equal number and horizontal diameter. The results obtained by simulation calculation were compared with that of the experiment to explore the magnetic field intensity distribution and the influence rule of diverse parameters on the magnetic flux.

## Conflict of Interest

The authors declare no conflict of interest.

## Supporting information

Supporting Information
