# Supplementary material for: A Material Combination Concept to Realize 4D Printed Products with Newly Emerging Property/Functionality
Source: Adv Sci (Weinh). 2020 Mar 20;7(9):1903208. doi: 10.1002/advs.201903208 (PMC7201257; doi:10.1002/advs.201903208)
Supplement: Supplementary file 1 — Supporting Information [file ADVS-7-1903208-s001.pdf]

Copyright WILEY-VCH Verlag GmbH & Co. KGaA, 69469 Weinheim, Germany, 2020.

ADVANCED SCIENCE
Open Access

Supporting Information

for *Adv. Sci.,* DOI: 10.1002/advs.201903208

A Material Combination Concept to Realize 4D Printed Products with Newly Emerging Property/Functionality

Hongzhi Wu, Xuan Zhang, Zheng Ma, Ce Zhang, Jingwei Ai, Peng Chen, Chunze Yan, Bin Su, and Yusheng Shi*

Supporting Information

A material combination concept to realize 4D printed products with newly-emerging property/functionality

Hongzhi Wu, Xuan Zhang, Zheng Ma, Ce Zhang, Jingwei Ai, Peng Chen, Chunze Yan, Bin Su, Yusheng Shi*

H. Wu, X. Zhang, Z. Ma, C. Zhang, P. Chen, Prof. C. Yan, Prof. B. Su, Prof. Y. Shi

State Key Laboratory of Materials Processing and Die & Mould Technology

School of Materials Science and Engineering

Huazhong University of Science and Technology

Wuhan 430074, P. R. China

Email: c_yan@hust.edu.cn

J. Ai

State Key Laboratory of Advanced Electromagnetic Engineering and Technology

School of Electrical and Electronic Engineering

Huazhong University of Science and Technology

Wuhan 430074, P. R. China

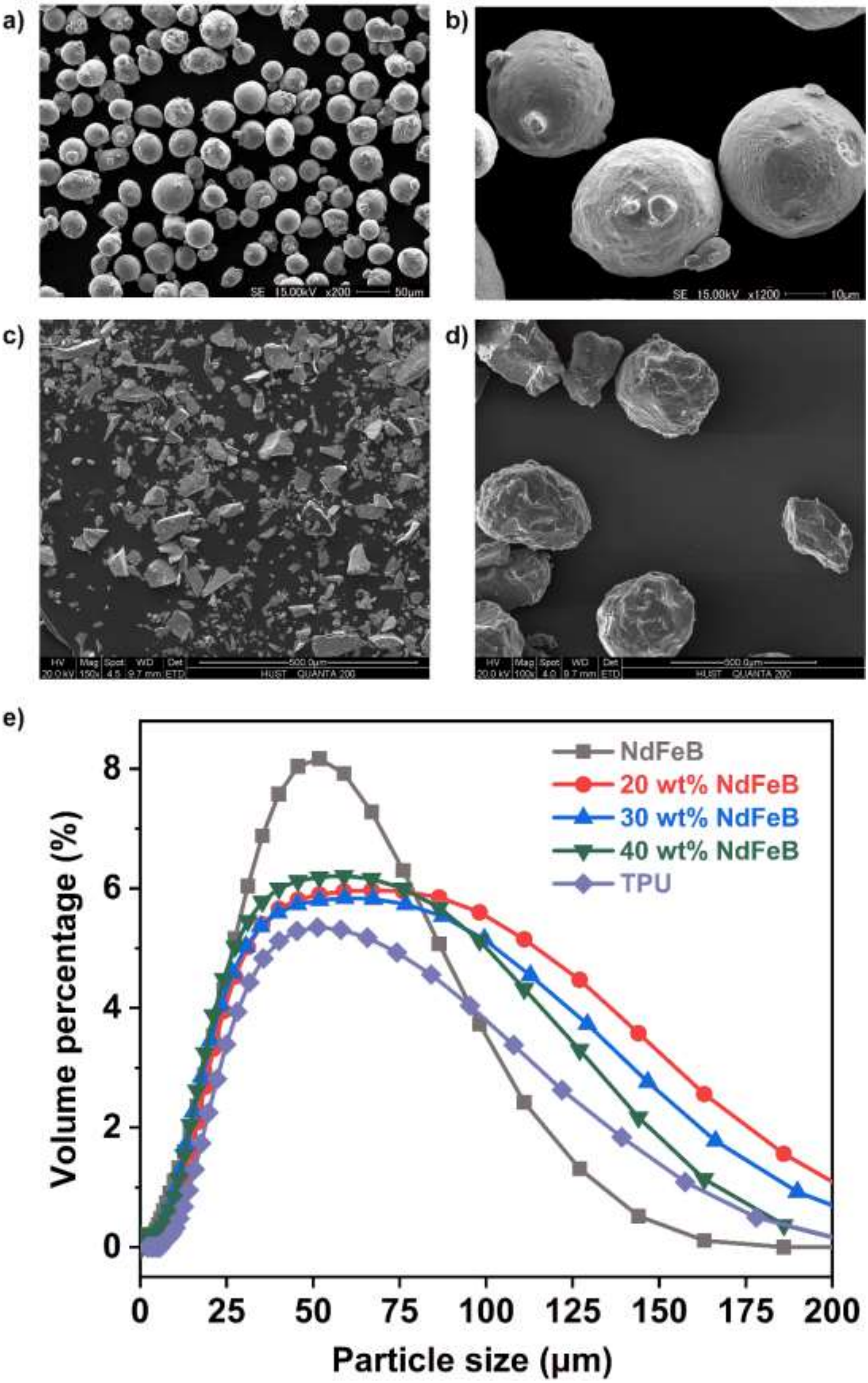

Figure S1 *The particle morphology of the 316L stainless steel powder, (a) 200 times magnification*

and (b) 1200 times magnification. The SEM images of (c) the NdFeB powder and (d) TPU powder. (e) Powder particle size distributions of the NdFeB, TPU and composite powders with three NdFeB contents.

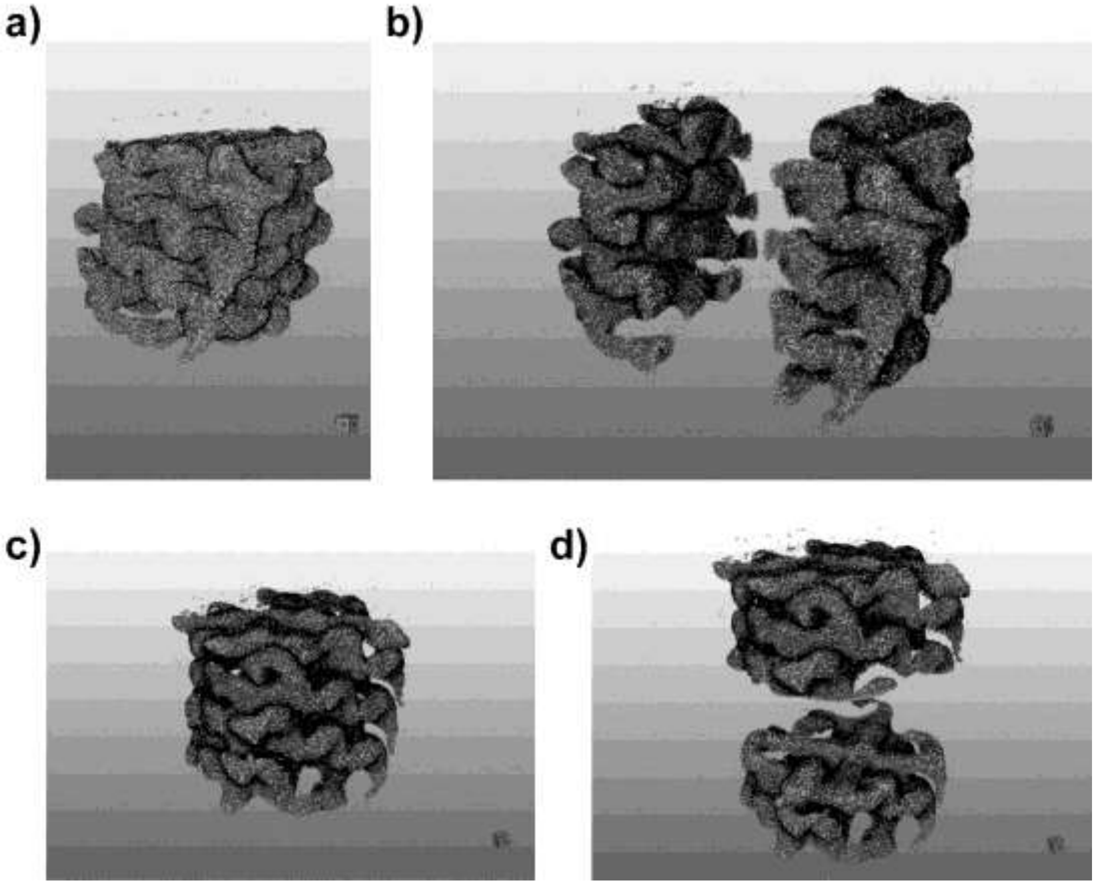

Figure S2 *The three-dimensional Micro-CT images of the magnetic porous structure under compression (a and c), which are vertically cut into (b) while horizontally cut into (d).*

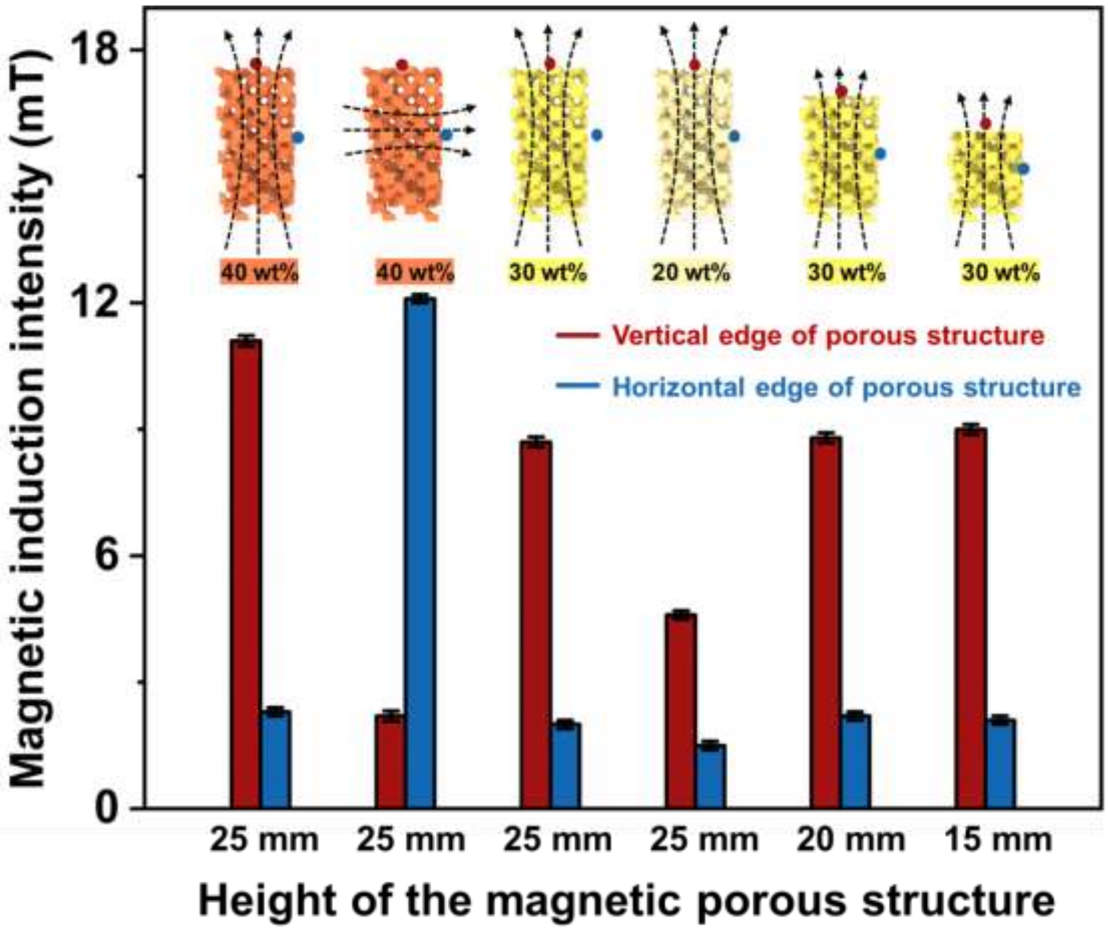

Figure S3 *The relationship between the magnetic induction intensity and NdFeB powder content, position and height of the magnetic porous structure. Red and blue points indicate the positions where the magnetic intensity was detected.*

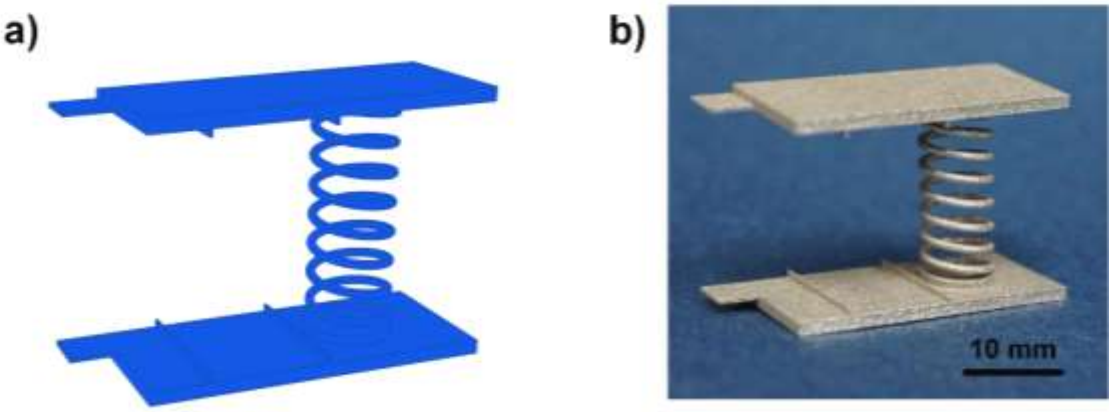

Figure S4 *(a) The computer aided design (CAD) model of the conductive helix with two flat plates and it has a height of 25 mm. (b) The optical photographs of the helix structure with a height of 25 mm fabricated by the selective laser melting (SLM) process.*

Note S1. Detailed simulation results of magnetic flux in the helix structure when the magnetic porous structure has vertical/ horizontal magnetization

1. Basic theory and simulation hypothesis

The Comsol finite element analysis software was applied to carry out the three-dimensional (3D) and two-dimensional (2D) modelling of a porous structure magnetized vertically and horizontally as well as before and after compression. The porous structure has the NdFeB content of 40 wt% and the size of 25×12.5×12.5 mm^3. In this study, the magnetic coercivity H_{cb} and residual flux density Br were set as 440.03 kA/m and 787.50 mT, respectively. For convenient calculation, the 7-layered helix structure considered as conducting coils was simplified into equivalent parallel rings (pink horizontal lines in Figure S5a, c and green horizontal lines in Figure S6a, c) with the same horizontal diameter and number.

In the simulation, it is assumed that the following conditions are valid:

(1) The 7-layered helix structure is simplified into equivalent seven parallel rings with the same horizontal diameter;

(2) The above-mentioned seven parallel rings are coaxial;

(3) The parallel rings are in an infinite vacuum.

As a result, the correlation between *z*-axis distance and the magnetic flux of before/after compression was illustrated in Figure S5b and (vertical magnetization), and Figure S6b and d (horizon magnetization), respectively.

According to Faraday's law of electromagnetic induction:

$$E(\mathrm{V}) = -N \bullet \frac{\Delta \Phi}{\Delta t} \qquad \textbf{(S1)}$$

where E is the generated output voltage, N is the number of rings, $\Delta\Phi$ is the total magnetic flux change, and Δt is the compression time of integrated 4D printed devices. As a consequence, the $\Delta\Phi$ and Δt can be expressed by Equation (S2)and (S3) shown as follows:

$$\Delta \Phi = \Phi_{after} - \Phi_{before} \qquad \textbf{(S2)}$$

$$\Delta t = t_{after} - t_{before} \quad \textbf{(S3)}$$

where Φ_{after} and Φ_{before} are the total magnetic flux after/before compression of integrated 4D printed devices, respectively. t_{after} and t_{before} are the moment when the device is after/before compression.

Owing to the nonuniform distribution of the magnetic field intensity around the magnetic porous structure, we should calculate the magnetic flux by its definition using two-dimensional surface integral. Thus the magnetic flux of one ring expressed as the following Equation (S4).

$$\Phi_i = \iint_{S_i} \vec{\boldsymbol{B}} \bullet \mathrm{d}\vec{\boldsymbol{S}} \quad \textbf{(S4)}$$

where $\vec{\boldsymbol{B}}$ is the vector form of the magnetic field intensity, $\vec{\boldsymbol{S}}$ is the vector form of the area of the ring, $\mathrm{d}\vec{\boldsymbol{S}}$ is the infinitesimal vector of the area, and the "•" between $\vec{\boldsymbol{B}}$ and $\mathrm{d}\vec{\boldsymbol{S}}$ is the dot product of vectors.

Then the total magnetic flux after/before compression can be expressed as below:

$$\Phi_{after} = \sum_{i=1}^{n} \Phi_{i,\ after} \quad \textbf{(S5)}$$

$$\Phi_{before} = \sum_{i=1}^{n} \Phi_{i,\ before} \quad \textbf{(S6)}$$

2 Vertical magnetization

According to Figure S5b and d, Φ_i is the point of intersection between the curve and the horizontal line representing the position of the ring. The $\Phi_{i,\ after}$ and $\Phi_{i,\ before}$ are listed in the following Table S1.

Table S1. *Calculated magnetic flux in the helix structure before/after compression when the porous structure was* ***vertically*** *magnetized*

The state of compression	The magnetic flux in each ring before and after compression ($\times 10^{-8}$ Wb)							
	1	2	3	4	5	6	7	**total**
$\Phi_{i,\ before}$	0.2	1.2	3.5	4.1	3.6	1.3	0.2	14.1
$\Phi_{i,\ after}$	0.9	2.8	4.1	4.6	4.2	2.9	0.9	20.4

$\boldsymbol{\Phi_{after}}$ - $\boldsymbol{\Phi_{befor}}$	6.3

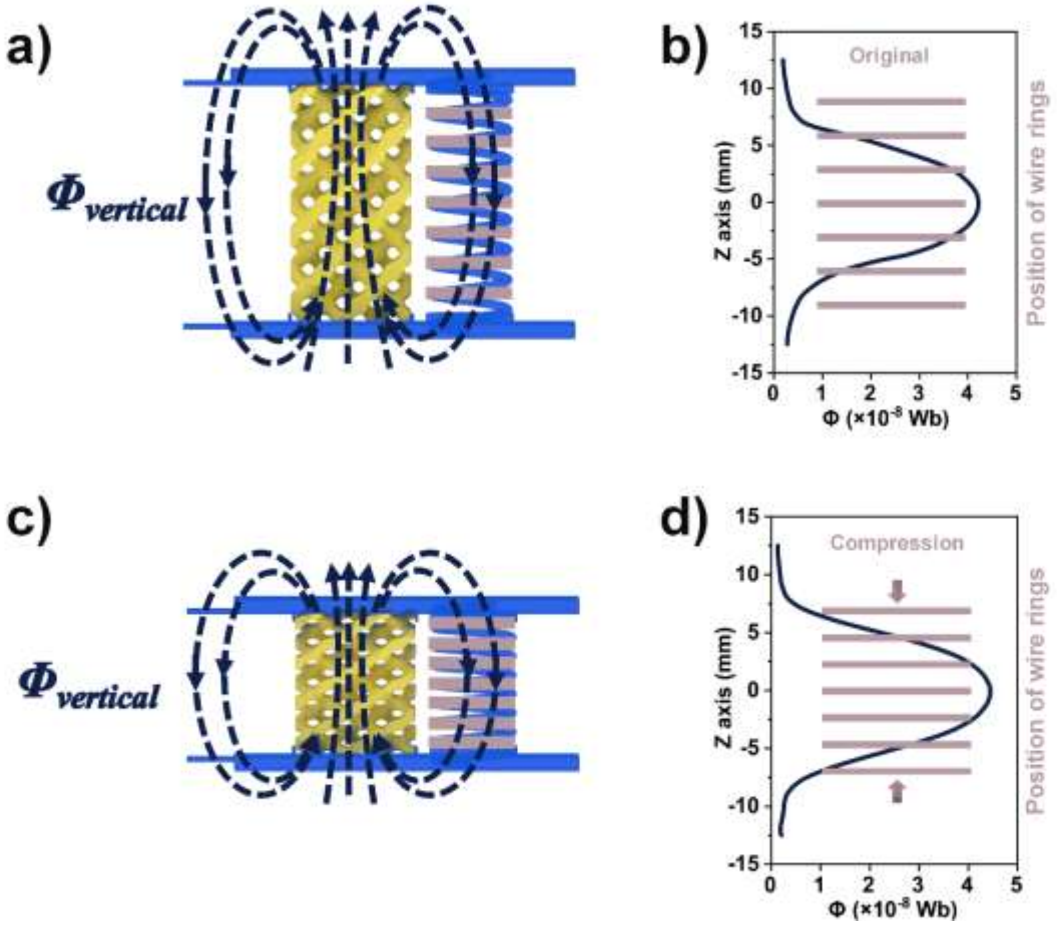

Figure S5 *The calculated magnetic flux of a vertically magnetized magnetoelectric device before and after compression. Schematic diagram of the direction of the magnetic induction line and the equivalent seven parallel rings (a) before and (c) after compression. The dependence of magnetic flux on the z-axis distance (b) before and (d) after compression based on numerical simulation.*

3 Horizontal magnetization

When the porous structure is horizontally magnetized, using the same analytical method, the $\Phi_{i,\ after}$ and $\Phi_{i,\ before}$ are calculated via Figure S6b and d and then listed in the following Table S2.

Table S2. *The calculated magnetic flux in the helix structure before/after compression when the porous structure was* ***horizontally*** *magnetized*

The state of compression	The magnetic flux in each ring before and after compression ($\times 10^{-8}$ Wb)							
	1	2	3	4	5	6	7	**total**

$\Phi_{i,\,before}$	4.2	4.7	1.6	0	-1.7	-4.8	-5.0	-1.0
$\Phi_{i,\,after}$	5.1	2.3	1.0	0	-0.8	-1.7	-3.9	2.0
Φ_{after} - Φ_{befor}								3.0

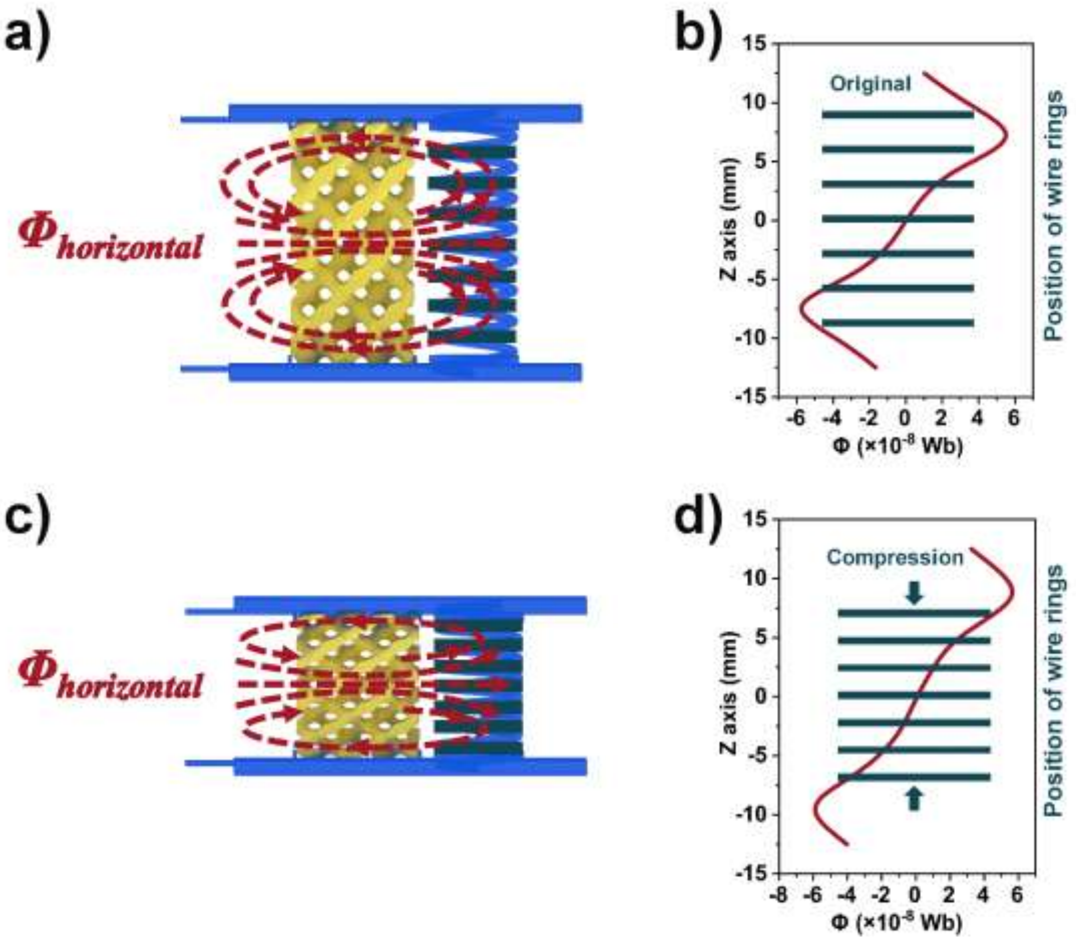

Figure S6 *The calculated magnetic flux of a horizontally magnetized magnetoelectric device before and after compression. Schematic diagram of the direction of the magnetic induction line and the equivalent seven parallel rings (a) before and (c) after compression. The dependence of magnetic flux on the z-axis distance (b) before and (d) after compression based on numerical simulation.*

Note S2. Detailed calculation of the average output voltage value of the experimental data by fitting the voltage curve using a sinusoidal function.

The results of numerical simulation often need to be compared with that of the experiment to verify the validity of the simulation. In this work, the output voltage value was obtained in these two methods. In an experimental way, the output voltage was recorded by an electrical measuring instrument and each data is an instantaneous value, so did the most noteworthy maximum value. While in the approach of numerical simulation, the output voltage value was calculated by Equation S1 as well as S2, …, and S6. In particular, special attention should be paid to the essential meaning of the E in Equation S1. It is an average output value, rather than an instantaneous one, during the compression process with an interval time of Δt. Consequently, it is not appropriate to directly compare the experimentally instantaneous values and theoretically calculated average values. Therefore, it is necessary to get the average value of the experimental results.

Due to a roughly sinusoidal shape of the experimental output voltage curve (Figure S7a), we chose a sinusoidal function to fit the curve, and calculated the average value using function theory.

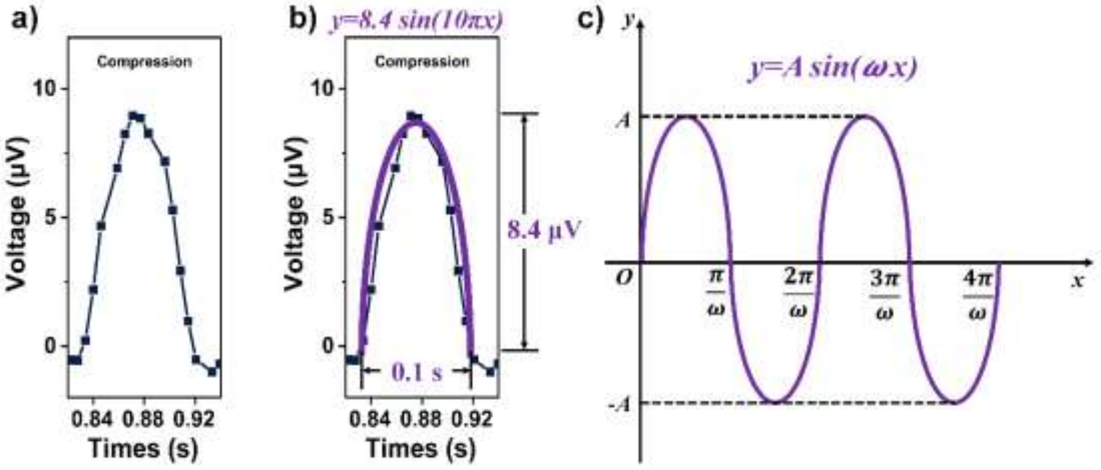

Figure S7 The experimental output voltage curve is fitted by a sinusoidal function. (a) The output voltage during a single compression cycle. (b) The schematic diagram of the experimental output voltage curve fitted by the curve (the purple line) of a sinusoidal function. (c) The function image of a sinusoidal function in general form.

On the basis of the trigonometric function theory, the function $y=A\sin(\omega x)$ has periodicity with period $T=2\pi/\omega$, and the maximum value is A. From the Figure S7b and c, it can be found that the compression time 0.1 s consists of a quarter period, so $\pi/\omega=0.1$, $\omega=10\pi$, and A is equal to the peak value of 8.4. Therefore, the chosen sinusoidal function is set to be $y=8.4\sin(10\pi x)$.

As for a function $y=f(x)$, its average value on the interval [a, b] is defined by Equation S7. So, the average value of the function $y=8.4\sin(10\pi x)$ between 0 and 0.1 can be calculated by Equation S8 and S9. We found that the average value only depends on the value of A.

$$\overline{f(x)}\Big|_{[a,\, b]} = \frac{\int_a^b f(x)\mathrm{d}x}{b-a} \quad \textbf{(S7)}$$

$$\int_0^{\frac{\pi}{\omega}} A\sin(\omega x)\mathrm{d}x = -\frac{A}{\omega}\cos(\omega x)\Big|_0^{\frac{\pi}{\omega}} = \frac{2A}{\omega} \quad \textbf{(S8)}$$

$$\overline{A} = \frac{\int_0^{\frac{\pi}{\omega}} A\sin(\omega x)\mathrm{d}x}{\frac{\pi}{\omega}} = \frac{2A}{\pi} \quad \textbf{(S9)}$$

According to the experimental results, the peak values of the output voltage were 8.4 µV and 4.4 µV when the porous structure was magnetized vertically and horizontally, respectively. The peak values of the output voltage for the vertical and horizontal magnetization were substituted to the Equation S9, and the corresponding average values can be figured out to be 5.3 µV and 2.8 µV, respectively.

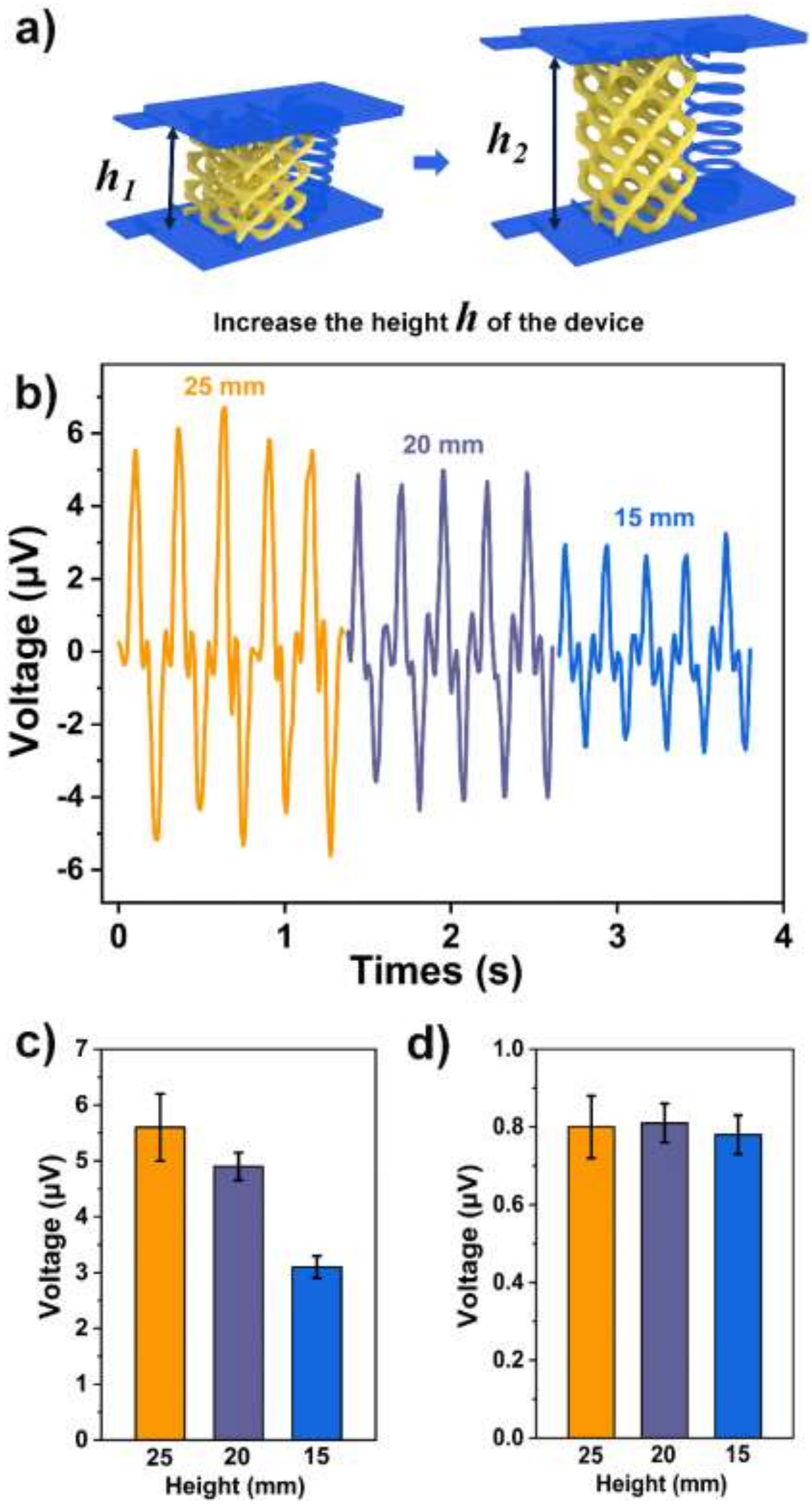

Figure S8 *The dependence of the device height on the output voltage of the 30 wt% NdFeB device when applied a cyclic compression with a strain of 20% and a speed of 50 mm/s. (a) Schematic illustration. (b and c) The output voltage of devices with three kinds of heights. (d) The output*

voltage generated by each coil.

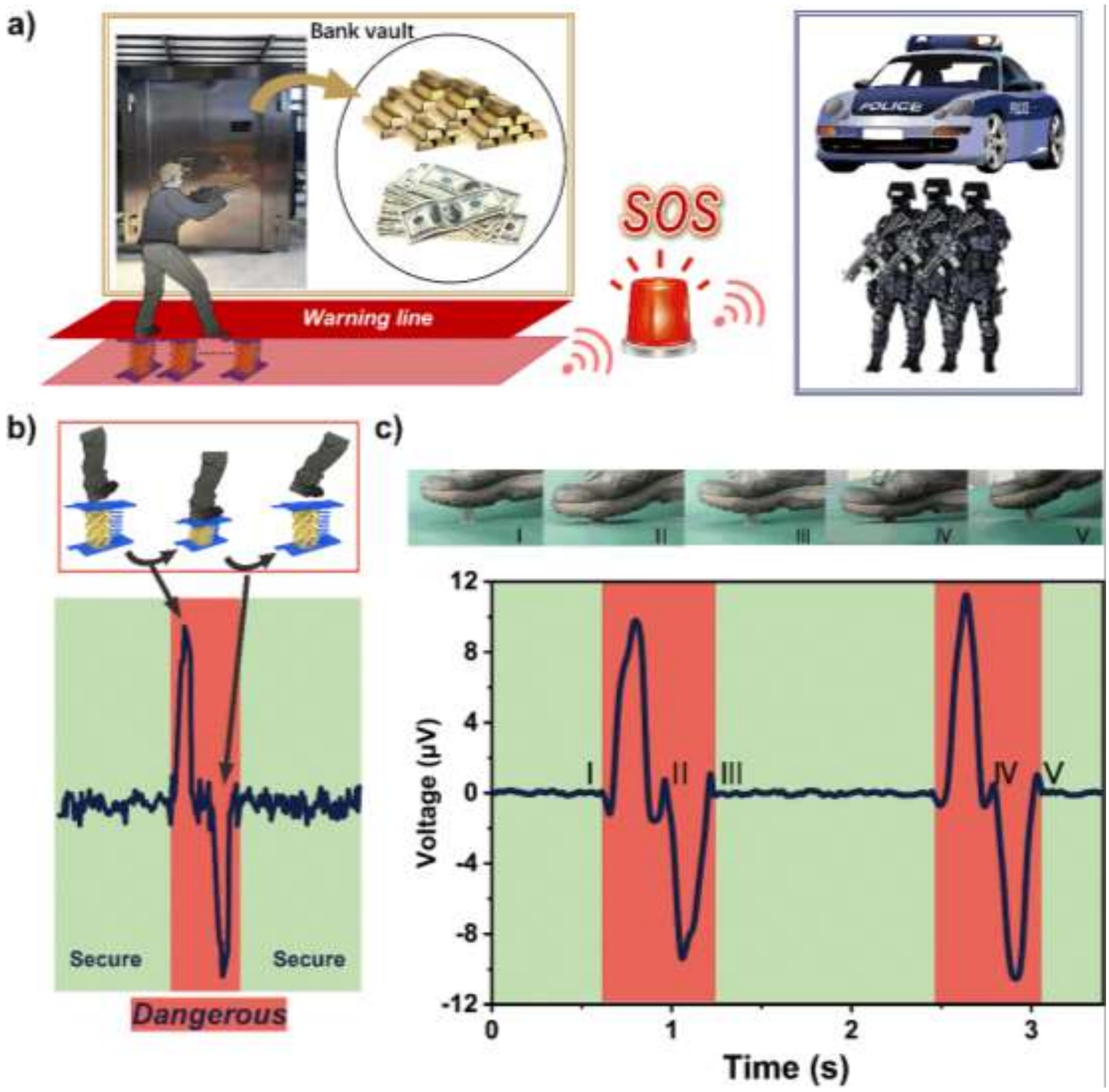

Figure S9 *Magnetoelectric devices based self-powered pressure sensors buried beneath the warning line of the bank vault. (a and b) Schematic illustrations. (c) Demonstration of the open-circuit output voltage of integrated 4D printed devices applied a cyclic compression/recovery by a person (can be considered as the gangster in (a)).*
